# A study of the influence of plasmonic resonance of gold nanoparticle doped PEDOT: PSS on the performance of organic solar cells based on CuPc/C6_0_

**DOI:** 10.1016/j.heliyon.2019.e02675

**Published:** 2019-11-14

**Authors:** D.A. Said, A.M. Ali, M.M. Khayyat, M. Boustimi, M. Loulou, R. Seoudi

**Affiliations:** a Physics Department, Faculty of Women for Art, Sciences and Education, Ain Shams University, Cairo, Egypt; b Department of Physics, College of Applied Science, Umm Al-Qura University, Makkah, Saudi Arabia; c Department of Physics, Faculty of Science, Mansoura University, Mansoura, Egypt; d King Abdulaziz City for Science and Technology, Riyadh 11442, Kingdom of Saudi Arabia; e Spectroscopy Department, Physics Division, NRC, Dokki, Cairo 12622, Egypt

**Keywords:** Materials science, Materials chemistry, Copper phthalocyanine, Gold nanoparticles, Fullerene, Solar cell, PEDOT:PSS

## Abstract

This work studied the role of gold nanoparticles (AuNPs) with different spherical sizes mixed with poly (3, 4-ethylene dioxythiophene): polystyrene sulfonate (PEDOT: PSS) as a hole transfer layer to enhance the efficiency (ITO/PEDOT:PSS (AuNPs)/CuPc/C_60_/Al) organic photovoltaic cell (OPV). AuNPs were synthesized using the thermochemical method and the results of the transmission electron microscope (TEM) images showed that the gold nanoparticles mostly dominated by spherical shapes and sizes were calculated in the range (12–23 nm). Measurements of UV-VIS spectra for AuNPs have shown that the surface plasmon resonance shifted to a higher wavelength with decreasing the particle size. Surface morphology and absorption spectra of OPV cells were studied using atomic force microscope and UV-VIS spectrometer techniques. The efficiency of the OPV cell was calculated without and with AuNPs. Efficiency was increased from 0.78% to 1.02% due to the embedded of AuNPs with (12 nm) in PEDOT/PSS. The increase in the light absorption in CuPc is due to the good transparent conducting of PEDOT:PSS and the increase in the electric field around AuNPs embedded in PEDOT:PSS and inbuilt electric field at the interfacial between CuPc and C_60_ is due to the surface plasmon resonance of AuNPs. The increase in these two factors increase the exciton generation in CuPc, dissociation at the interfacial layer, and charge carrier transfer which increases the collection of electrons and holes at cathode and anode.

## 1 Introduction

Gold nanoparticles prepared in the range (1–100 nm) have distinct optical and physical properties depending on their size and shape [1, 2, 3]. The characteristic optical properties are due to the absorbance and scattering of incident light at Localized Surface Plasmon Resonance (LSPR). The LSPR is the collective oscillation of electrons in the conduction band in resonance with the incident-specific wavelength [4]. The band absorbance of LSPR increases with increased particle size. Colloidal gold nanoparticles are synthesized by reduction of citrate [5, 6, 7, 8]. In reduction methods, the particle sizes and shapes can be controlled by changing the reaction temperature or the concentration of the reducing agent [9, 10, 11, 12]. More recently, gold nanoparticles have been used in technological applications such as organic photovoltaic cells [13, 14]. Organic semiconductor solar cells have gained widespread attention in recent years due to low production costs, ease of fabrication, flexibility and tunable optical properties. Furthermore, phthalocyanine (Pc) as an organic semiconductor has been widely used as attractive materials for photovoltaic cells [15, 16]. Copper phthalocyanine (CuPc) is one of the most important organic molecules useful for thin organic solar cells [17, 18]. Organic solar cells (OSCs) are suitable for solar light harvesting due to both properties and preparation process. Transparent conductive films (TCF) are materials that must fulfill the requirements of high conductivity and high optical transparency for use in various applications of solar cell device as an anode. Graphene [19], conductive polymer [20], metal nanowires [21], aluminum zinc oxide [22], metal nanostructures consisting of nanowires, nanogrid, nanofibres [23], and carbon nanotubes (CNT) [24, 25] have been applied as transparent conducting films (TCF). Chakaroun et. al. made three-layer anodes of ITO/Au/ITO in CuPc/C_60_ photovoltaic cells to achieve low resistivity of the anode and to improve the fill factor and efficiency [26]. It can be replaced ITO by PEDOT:PSS as a front electrode in organic solar cells because of its high conductivity, transparency and work function in the range of 5.1 eV which makes it suitable as an anode. metal grids can be used on the PEDOT:PSS film [27, 28, 29]. In our work, indium tin oxide (ITO) is used as TCF (anode) due to an excellent characteristic of the low sheet in the range of 10–25 Ω/sq. and its high optical transmittance (T) greater than 85 %. Peumans and Forrest used bathocuproine (BCP) as an electron transport layer (light absorber) to disturb the diffusion of excitons to the cathode and to decrease the later exciton quenching at the acceptor/cathode interface in the (CuPc/C_60_) OPV cell [30]. Different materials can be used as a transport layer such as bathophenanthroline (BPhen), 1,3,5-tris (N-phenylbenz-imidazol-2-yl) benzene (TPBi), and tris(8-hydroxyquinolinato) aluminum (Alq3) [31]. In the other side, Poly(3,4-ethylene dioxythiophene): poly(styrenesulfonate) (PEDOT: PSS), is an attraction of photoconductor transport layer due to its high transparency in the visible range, long-term stability, easy processing, and nontoxicity [32]. So, PEDOT: PSS has become an excellent choice for interfacial/electrode material and hole transport layer in many applications, such as organic solar cell OSCs [33, 34]. Groenendaal et al. [35], Kirchmeyer and Reuter [33] succeed in fabricating transparent flexible electrodes from poly(3,4-ethylene dioxythiophene) (PEDOT) and poly(styrenesulfonate) (PSS), doped by polythiophenes. Shin et. al. [36], improved performances of an OPV cell by an embedded poly(3,4-ethylenedioxythiophene):poly(styrene sulfonate) (PEDOT:PSS) by the larger size of AuNPs (71, 80, 87, 103 nm). The improvements in efficiency were attributed to the near-field coupling localized surface plasmon resonance (LSPR) at the size of AuNPs smaller than 80 nm and far-field scattering with sizes greater than 87 nm. Dutta et. al., embedded PEDOT:PSS buffer layer by Eu3+ to enhance the photon concentration by converting UV light to the visible spectrum [37]. Soga et. al., also improved the efficiency of the organic solar cell by doping aluminum microstructures in (PEDOT:PSS) buffer layer and observed an increase in both current density, and fill factor due to due to increase of the transport charge carriers [38]. Wei et. al., enhanced the power conversion efficiency of OPV cell by depositing a layer of copper iodide on the PEDOT:PSS as hole transport. The electron and hole extraction in the donor-acceptor layer was increased by photoluminescence spectra [39]. Zhang et. al. used a random and irregular garnish of (PEDOT: PSS)/CuPc/C_60_ as an anode buffer layer, donor and acceptor, respectively. The irregular garnish nanostructure with a random distribution of PEDOT/PSS in the OPV increases the junction area, light paths, light absorption, short-circuit current, fill factor and efficiency due to increase in the exciton dissociation, charge carrier transfer, and collection in the active layer by scattering of the incident light [40]. The phase separation in CuPc:C_60_ film with an improved ratio of the corresponding electronic absorption and carrier transport which are necessary for the performance of OPV cell were studied [41]. Rezaei et al., have enhanced the efficiency of polymer solar cells by incorporating gold/graphene core-shell nanoparticles between hole transport and active layers. The roughness to the active layer, optical absorption, and charge transfer has been improved by the core shell. Graphene shell over AuNPs improved the lifetime of the charges 80 %, short circuit current (42%) and efficiency (100%) compared to reference [42]. Kim et. al. compared the efficiency of OPV cells and found that they changed from 1.28% and 1.31% when using a new style of forming Ag back electrode forming using a bonding process instead of a conventional screen-printed Ag. The binder resin at the interface between the PEDOT:PSS and the Ag may increase contact resistance for the screen-printed Ag process [43]. Yu et. al. studied the effect of HAuCl4 and Au nanoparticles (NPs) concentration and annealing temperature on the properties of PEDOT:PSS/PTB7:ICBA. An enhancement of the light absorption in the polymer solar cells by extinction PL spectra from Au NPs doping in PEDOT:PSS layer because of localized surface plasmon resonance (LSPR). An improvement in power conversion efficiency was observed compared with the reference device without HAuCl4 doping. Significant improvement was sawed at PTB7:ICBA system with a high PCE of 8.05%, compared with the reference device without HAuCl4 doping (5.88 ± 0.05%) [44]. Zhang and Holmes used a rubrene as an insulating blocking interlayer at (CuPc) donor-C_60_ acceptor interface to disappointed recombination losses and to generate a separate of electron and hole. The interlayer between rubrene and CuPc can permeable excitons and this led to an increase in open-circuit voltage, short-circuit current, and power efficiency [45]. In this work, PEDOT: PSS is embedded with gold nanoparticles of different sizes and deposited on a conducting glass (Glass/ITO) as a thin film to use a hole transport layer for the organic solar cell. These organic solar cells rely on the thin film of CuPc/C_60_ as a donor and acceptor, respectively. The AuNPs and organic solar cell doped by AuNPs with different sizes are characterized by TEM, Uv-Vis spectrometer, and AFM. The effect of Au nanoparticle sizes on the power conversion efficiency was investigated to achieve the optimum conditions for increased efficiency.

## 2 Experimental

### 2.1 Chemicals

Tri-sodium citrate dehydrate (Na_3_C_6_H_5_O_7_.2H_2_O), tetrachloroauratetrihydrate (HAuCl_4_·3H_2_O, 99.9%), Fullerene (C_60_, 99.5%), poly(3,4-ethylene dioxythiophene) polystyrene sulfonate (PEDOT:PSS) 1.3 wt.% dispersion in H_2_O conducting grade and copper (II) phthalocyanine were purchased from Sigma-Aldrich (USA) and used as received without further purification. Distilled water is used as a solvent in all preparation processes. Conducting Glass (Glass/ITO) purchased from Kintec Company (Hong Kong) were sonicated using ultrasonic bath with acetone, rinsed with deionized water and dried under vacuum. ITO thin film has a resistivity of 20 ohm/sq.

#### 2.1.1 Sample preparations

The colloidal forms of gold nanoparticles were prepared by adding 1.75 mL of 1% sodium citrate solution to 20 ml of HAuCl_4_.3H_2_O (1.0 mM) boiling solution under magnetic stirring. Sample (S_1_) was removed when the solution became dark red. This process was repeated by adding different molar ratios (1.5, 1.25, 1, 0.75 and 0.5 mL) of sodium citrate solution 1% to 20 ml of HAuCl_4_ (1.0 mM) boiling solution under magnetic stirring to prepare samples (S_2_-S_6_).

#### 2.1.2 Spin coating of PEDOT: PSS mixed by gold nanoparticles

**Fig. 1:**
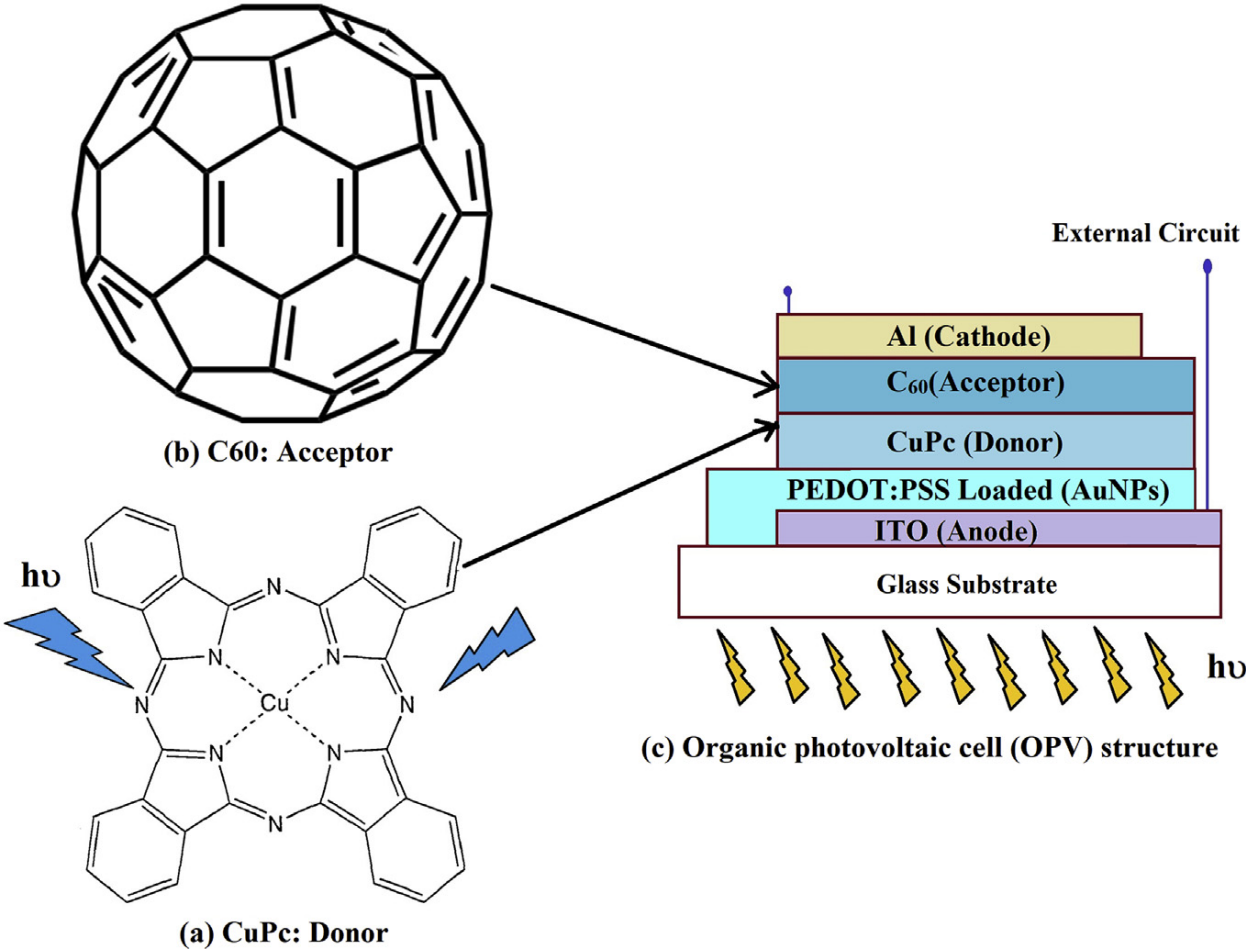
(a) chemical structure of copper phthalocyanine CuPc as a donor, (b) fullerene (C_60_) as acceptor, and (c) schematic structure of the organic photovoltaic cell.

To prepare a composite of a hole transfer layer PEDOT:PSS embedded by AuNPs, the same volume ratio of AuNPs solution (S1-S6) was mixed with PEDOT: PSS solution separately. The volume ratio of AuNPs solution to volume ratio of PEDOT:PSS solution was 10%. A thin layer of PEDOT: PSS mixed with AuNPs was spin-coated on conducting glass plates at 3500 rpm for 50 seconds under vacuum at room temperature. Thin films were annealed at 85 °C for 2 hr. to evaporate the excess of water molecules from the samples and to increase the adhesion between composites films and ITO on a glass substrate. CuPc and C_60_ with a chemical form as shown in Fig. 1: a, b ​as a p-type and n-type thin films were prepared on (Glass/ITO/PEDOT:PSS) and on (Glass/ITO/PEDOT:PSS:AuNPs) by the conventional thermal evaporation technique, using a high vacuum coating unit (Edwards type E 306 A, England) at a pressure of about 1.7×10^−5^ Torr with the thickness of 65 nm and 70 nm respectively. Aluminum (Al) contacts with a thickness of 80 nm were evaporated as a top electrode. A schematic diagram of the present solar cells is shown in Fig. 1: c.

### 2.2 Samples characterization and measurements

Morphology and distribution of the AuNPs samples were studied using TEM (JEOLJEM-1011; Japan) operating at an accelerating voltage of 120 kV. TEM samples were prepared by depositing one drop of colloidal AuNPs onto a standard carbon-coated copper grid (300 mesh) and allow it to dry before doing the TEM measurements. UV-VIS absorption spectra for both the AuNPs and thin films of CuPc, C_60_, and (ITO/PEDOT:PSS embedded by AuNPs with different sizes/CuPc/C_60_/Al) films were measured at room temperature using a spectrophotometer (Thermo-scientific Evolution 220) at a resolution of 2 nm. The incident light was made from the glass substrate side. Surface morphology of (ITO/PEDOT:PSS embedded by AuNPs with different sizes/CuPc/C_60_/Al) films was measured using AFM tool (NTEGRA Probe Nano Laboratory from NT-MDT) controlled by the computer software of the Control Program Nova Px. The images were obtained, in tapping mode, with HA_NC A probe (force constant 12 N/m and a resonant frequency 235 kHz). The current density-voltage (J-V) characteristic of the junctions was measured under illumination at 100 mW cm^2^ by using the experimental setup consists of Keithley electrometer 6517 A, DC power supply.

## 3 Results and discussions

### 3.1 TEM of gold nanoparticles

**Fig. 2:**
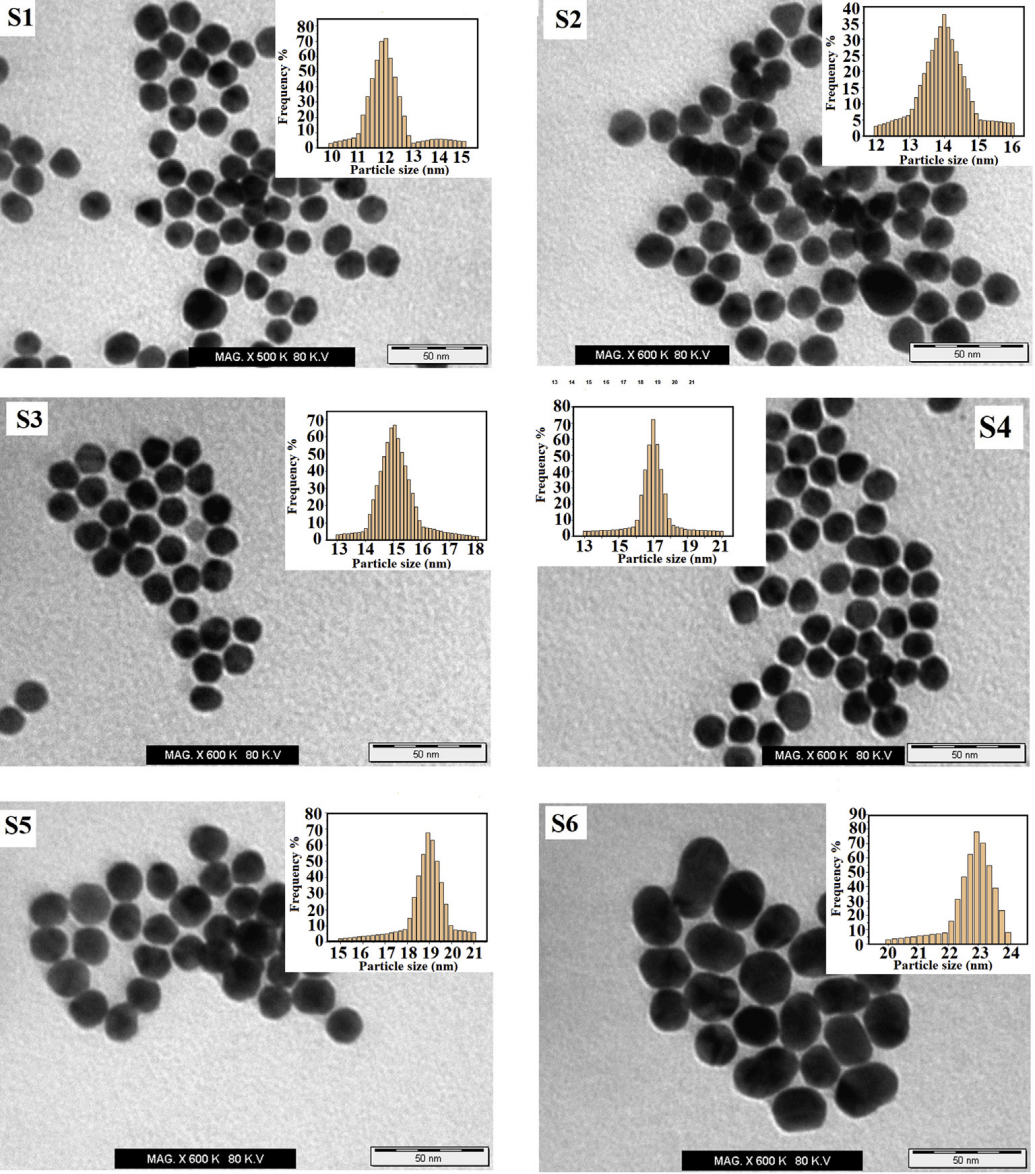
Transmission Electron Microscope of gold nanoparticle samples (S1-S6) prepared by adding of (0.5, 0.75, 1.0, 1.25 1.5, and 1.75 ml) from tri-sodium citrate (1%) to boiling of 20 ml of HAuCl_4_·3H_2_O (1.0 mM), respectively.

TEM has been achieved to verify the shapes, size distribution, and to estimate the particle size of the dispersed suspension of AuNPs. Fig. 2 shows the TEM images of AuNPs samples (S1-S6) that prepared by adding of (0.5, 0.75, 1.0, 1.25 1.5, and 1.75 ml) from tri-sodium citrate (1%) to boiling of 20 ml of HAuCl_4_·3H_2_O (1.0 mM) respectively. Small dark particles were observed in TEM images due to the presence of AuNPs. Most of the particles are spherical in shape and in the monodispersion state. Particle sizes calculated by measuring the diameter of whole particles of TEM images. The average diameter of all AuNPs calculated at (12, 14, 15, 17, 19, and 23 nm) as shown inset of the Fig. 2 with a few higher and lower size distribution of (S1-S6) respectively. The TEM analysis of the AuNPs was consistent as expected for in general, behavior, the size increased with increased the molar ratio of trisodium citrate. In general, it observed that the particles size evolution of the formed AuNPs increased by increasing the tri-sodium citrate and display large hydrodynamic diameter (23 nm) at 1.75 mL of citrate and decreasing the size and reaching the minimum diameter (12 nm) at 0.5 mL of citrate as well as reaching the plateau at 0.75 and 1 mL of citrate. The AuNPs increased with increasing the citrate due to the total electrolyte concentration is enough to curdle of the nanoparticle, which may lead to increase in the size. Although the presence of few aggregates can be detected in high molar of citrate. These results are consistent with some of the finding reported by Kimling et al. [46] and Zabetakis et al. [47].

### 3.2 Ultraviolet-visible spectroscopy

**Fig. 3:**
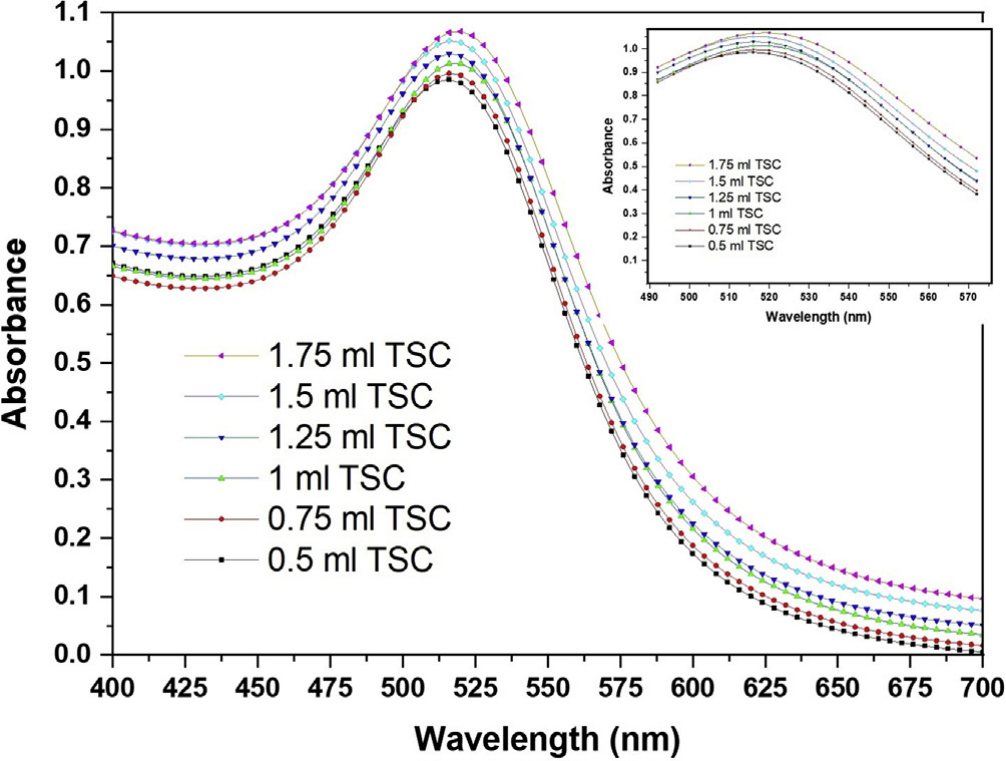
UV-Vis. spectra of gold nanoparticle prepared by adding different molar ratios of trisodium citrate (TSC) to 20 mL boiling of HAuCl_4_·3H_2_O (1 mM).

Fig. 3 shows the UV–visible spectra of all dispersion of spherical AuNPs samples (S1-S6) prepared by adding different molar ratio of tri-sodium citrate (TSC) (0.5, 0.75, 1.0, 1,25 1.5, and 1.75 ml) to 20 mL boiling HAuCl_4_ (1.0 mM). The analysis of the absorption spectra revealed a centered band were appeared at 512, 514, 515, 517, 519, and 523 nm for the samples (S1-S6), respectively. These bands originated from absorption photons and are interpreted as surface plasmon resonance band (SPR) of AuNPs. SPR is appearance due to the excitation of the electron cloud at the incident electromagnetic wavelength is more than the AuNPs sizes. In the case of resonance, the amplitude of the local electric field in the particle $E_{\ell }$ intensified in comparison with the one of applied fields $E_{o}$. In other words, the complex local field factor $f_{\ell }=E_{\ell }/E_{o}$ greater the SPR [48, 49]. It can be note that the position of these bands is dependent on the particle sizes of AuNPs. The maximum band position was shifted to the higher wavelength (red shift) with increasing the particle sizes which increased by increasing TSC molar ratio.

### 3.3 UV-Vis spectra (Glass/ITO/(PEDOT:PSS:AuNPs)/CuPc/C_60_) thin films

**Fig. 4:**
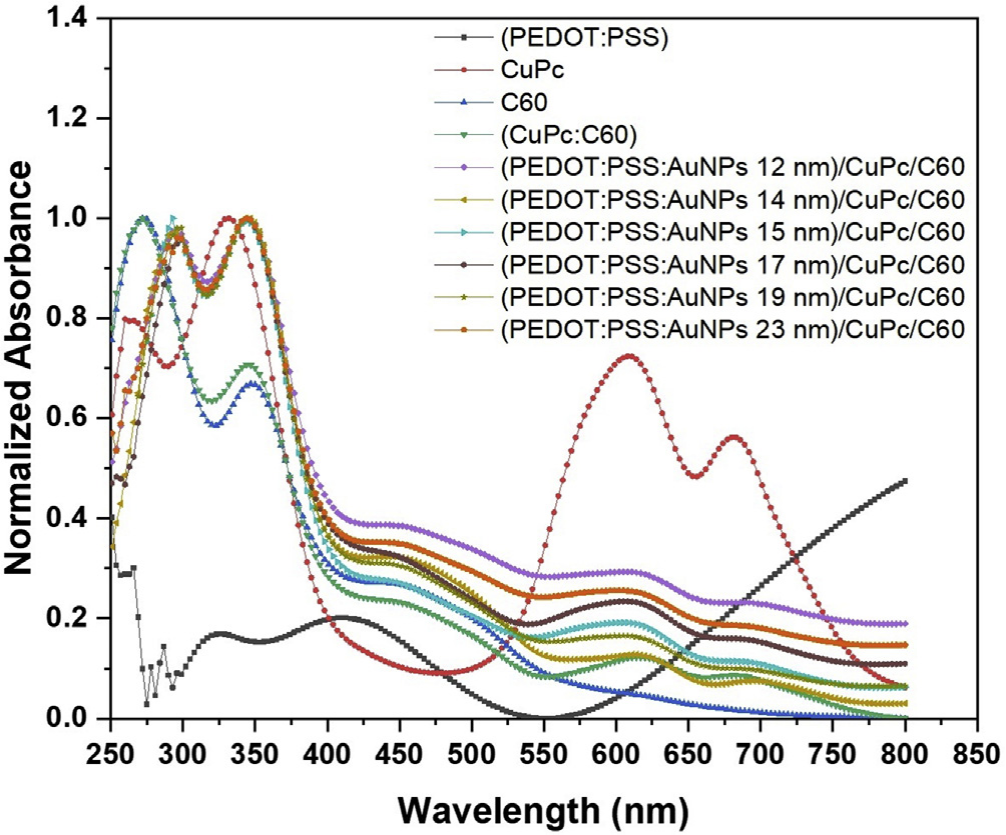
Normalized absorption spectra of CuPc, C60, different sizes of gold nanoparticle embedded in PEDOT: PSS/CuPc/C60 multilayer films.

PEDOT: PSS is used in organic solar cells as a photoconductor transport layer and as an electrode which is one of the factors affect the efficiency of the device. Fig. 4 shows the normalized absorption spectra of CuPc, C_60_, and (ITO/PEDOT:PSS embedded by AuNPs with different sizes/CuPc/C_60_/Al) films. From the spectrum of the PEDOT/PSS, it is seen that PEDOT: PSS has two small absorption bands at about 415 nm and 320 nm. These two bands referred to the π–π* transition and are assigned to thiophene ring in PEDOT [50] and benzene rings in PSS, respectively [51]. The absorption value was increased after 700 nm. CuPc as an organic semiconductor thin film has three absorption bands at about 340, 595, and 685 nm. The first band UV- region (B- band region) is due to the highly conjugate of the ring and the n-electrons cloud formation of the molecule in a ligand [52]. The last two bands (Q-band region) are assigned to the metal-free phthalocyanine (Pc).

In addition, these two bands interpreted the first electronic transitions $a_{1u}$ to $2e_{g}$ $\left(\pi \to \pi^{*}\right)$ and second electronic transitions $a_{2u}$ to $2e_{g}$ $\left(\pi \to \pi^{*}\right)$, respectively [53]. These bands correspond to the intermolecular excitation coupling between the aromatic cores of neighboring phthalocyanine macrocycle. The intensity of the transitions band at 685 nm is weak due to the poor orbital overlap of the end. The spectrum of the C_60_ thin film in Fig. 4 shows the absorption peaks at about 350 nm (3.54 eV) and 450 nm (2.75 eV). According to the orbital model of molecules, these two bands are corresponding to the (HOMO→LOMO)dipole transition $\left(H_{u}\to T_{1g}\right)$ and $\left(H_{1g}\to T_{1u}\right)$ [54]. PEDOT:PSS, C_60_, and CuPc thin films spectrum were compared with (PEDOT:PSS embedded by different AuNPs/CuPC/C_60_). No new bands were observed but the value of the absorbance band was increased in the range (510–650 nm). Furthermore, there is no absorption band assigned to the gold nanoparticles indicating the low distribution of AuNPs in the film.

### 3.4 Atomic force microscope resuts of (glass/ITO/PEDOT: PSS: AuNPs/CuPc/C_60_) thin films

**Fig. 5:**
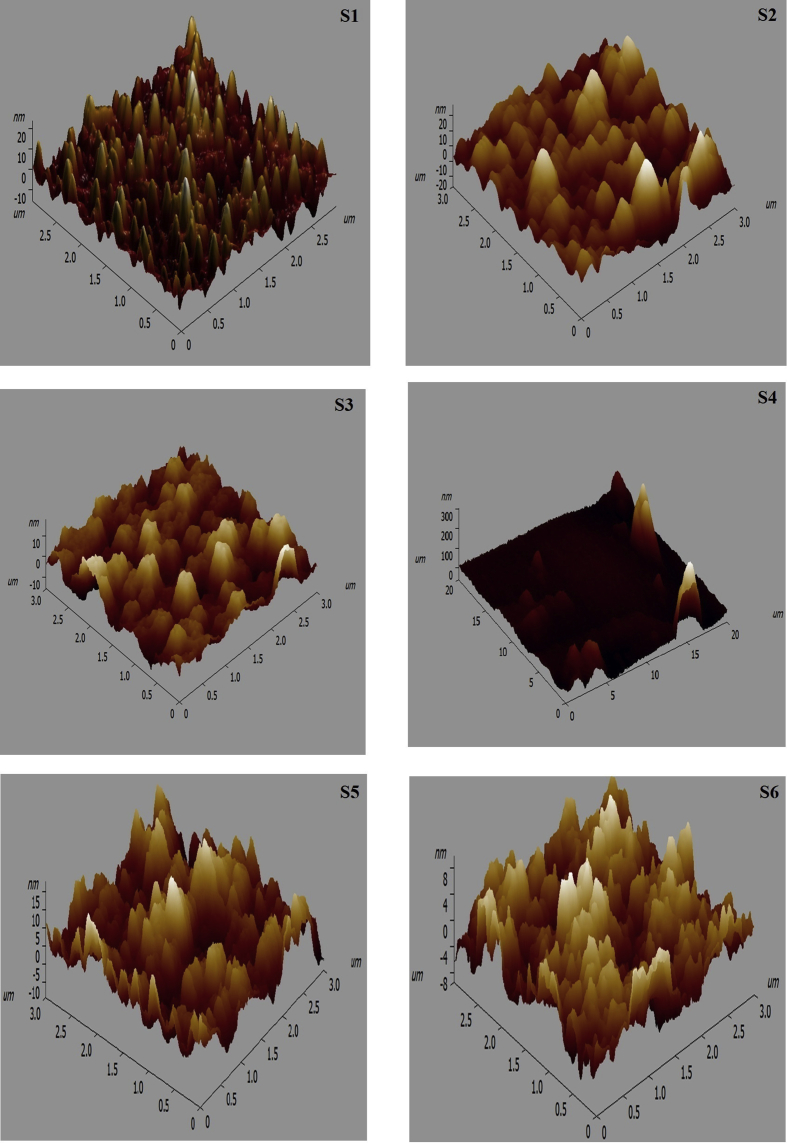
AFM images of gold nanoparticles samples (S1-S6) that prepared by adding of (0.5, 0.75, 1.0, 1.25 1.5, and 1.75 ml) from tri-sodium citrate (1%) to boiling of 20 ml of HAuCl_4_·3H_2_O (1.0 mM), respectively and doped in PEDOT: PSS/CuPc/C60 multilayer films on ITO/glass substrate.

Fig. 5 shows AFM images of PEDOT: PSS embedded by AuNPs samples (S1-S6) which synthesized by adding of (0.5, 0.75, 1.0, 1.25 1.5, and 1.75 ml) from tri-sodium citrate (1%) to boiling of 20 ml of HAuCl_4_·3H_2_O (1.0 mM) respectively/CuPc/C_60_ thin films. Some irregular distribution of nano-holes (as bowel shaped) is observed for each sample. In addition, different dimensions, roughness (as conic) and surface morphology appeared in all films. Changes of the embossed feature for each sample alone may be due to the change of AuNPs particle sizes in PEDOT: PSS film. Individual AuNPs agglomerates on the PEDOT: PSS increases the height, concave and convex of the conic shapes compared to the average film thickness and increases surface roughness. The existence of AuNPs on the film surface was discovered by the scanning tip. Table 1 shows the estimated parameters as the root mean square roughness, average roughness, maximum peak height, and maximum valley depth in nanometer range of all samples. It was found that the root-mean-square roughness was calculated at 35.6 nm of the sample S4 which indicates that the film quality was affected by adding AuNPs with size 17 nm, but not affected by the last sizes of AuNPs samples. This is probably during the preparation of the PEDOT:PSS embedded with AuNPs (17 nm) using spin coater some aggregation has happened from these samples that appeared increasing in the roughness of the film. It can be expected that the roughness of the film without AuNPs is less than 2.869 (less than of roughness value) in Table 1.

**Table 1:** The surface parameter of gold nanoparticles samples (S1-S6) that prepared by adding of (0.5, 0.75, 1.0, 1.25 1.5, and 1.75 ml) from tri-sodium citrate (1%) to boiling of 20 ml of HAuCl_4_·3H_2_O (1.0 mM), respectively and doped in PEDOT: PSS/CuPc/C60/ITO/glass substrate.

AFM parameters	S1	S2	S3	S4	S5	S6
Root mean square roughness (nm)	5.896	06.197	4.7590	35.5660	4.5310	2.869
Average roughness (nm)	4.673	5.3330	3.6850	19.0710	3.6540	2.281
Maximum peak height (nm)	24.820	27.398	18.053	306.669	17.996	9.199
Maximum valley depth (nm)	15.733	25.567	15.523	61.8840	14.157	8.106

### 3.5 Generation and dissociation of excitons

**Fig. 6:**
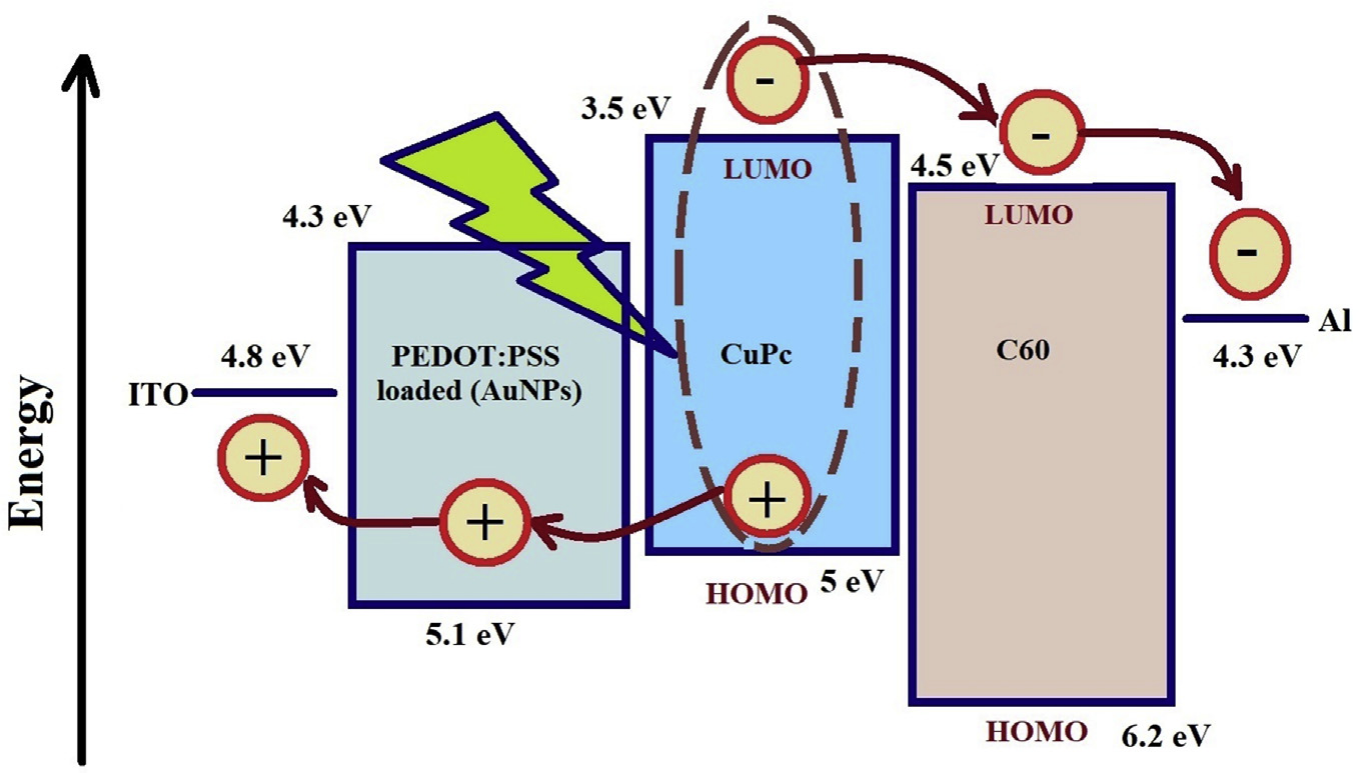
Photogeneration and dissociation of exciton (electron hole pairs).

The basis of the working of plasmonic solar cells contains absorption of light and surface plasmonic resonance due to the embedded of AuNPs in PEDOT:PSS. The light incident on the PEDOT:PSS embedded by different sizes of AuNPs passes through the thin ITO that does not absorb the light as shown in Fig. 6. This incident light passes to CuPc (donor) to generate excitons that can be diffused in CuPc layer or dissociated at the interface layer between CuPc and C_60_. The dissociation occurs when the time of excitons diffuses more than the time of decomposition of the excitons. As we know, the exciton has a binding energy and it needs the energy to dissociate so the built-in electric field resulting from the difference in work function between CuPc (donor) and C_60_ (acceptor) or the electrostatic forces that generated due to the difference between the electron affinity and ionization potential for both C_60_ and CuPc has potential energies overcome excitonic binding energy. So, it is suitable for dissociate photogenerated excitons at the donor-acceptor interface. To improve the efficiency of OPV cell, we need to increase both the number of excitonic generation in CuPc and the field near the interface between CuPc and C60 to increase the exciton dissociation. It has been found that PEDOT:PSS is a good a transparent conducting layer so it passes the incident light to CuPc layer in order to increase the generation of the (e-h) exciton in CuPc and surface plasmon resonance of AuNPs doped in PEDOT:PSS has increased the electric field around the AuNPs. This electric field enhances the built-in electric field at the interface between CuPc and C60 that contributes to an increase in the decomposition of excitons. This technique is very useful for OPV due to their diffusion length is short. Thus, the embedding of AuNPs in PEDOT:PSS is beneficial to increase the densities of excitons that corresponds to the resonance. We used AuNPs due to it is highly stable, has broader resonance peak, and surface resonance frequency depends on the free electron density in the particle. These results are consistent with previous work done [55, 56, 57, 58, 59].

### 3.6 The current density–voltage

**Fig. 7:**
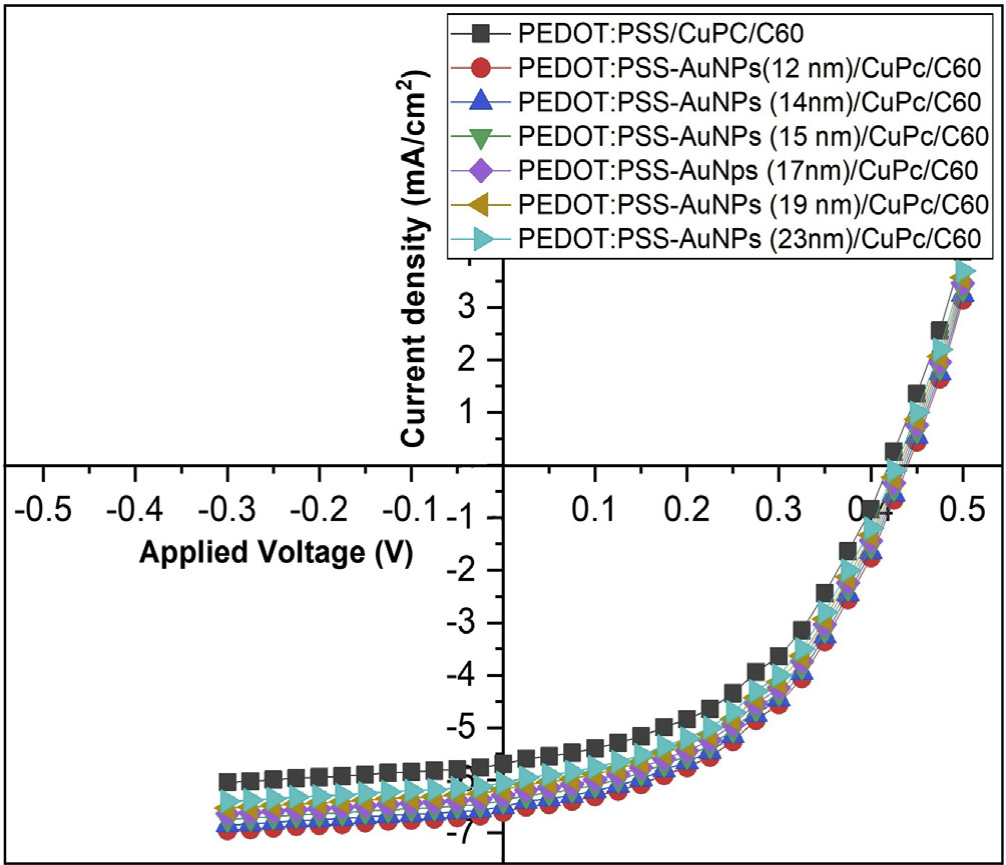
Current density (J) ∼Voltage (V) characteristics of organic solar cells with Structures ITO/PEDOT:PSS:AuNPs (with different sizes)/CuPc/C60/Ag, AM 1.5G illumination at 100 mW/cm^2^.

Enhanced photovoltaic achievement can be assigned to the plasmonic effect of nanoparticles and the rapid generate of excitons at CuPc as a donor layer and decomposition at the interfacial layer between donor and C_60_ as acceptor. In this phenomenon, the light is further intensified into the donor layer to be absorbed in CuPc and increase the built-in field at the interfacial layer due to PEDPT:PSS embedded AuNPs and to the surface plasmonic resonance effect. The surface plasmonic resonance is known as the oscillation of electrons in nanoparticles which was created by the interaction between the incident light and the surface of nanoparticles. Resonance condition occurs when the frequency of the incident photons matches with the natural frequency of the surface metallic electrons against the positive nuclei restoring force. The optimization of the AuNPs in the PEDOT:PSS is important to improve the performance of CuPc/C_60_ solar cell. (J–V) characteristics of ITO/PEDOT:PSS embedded by different sizes of AuNPs/CuPc/C_60_/Al solar cells are presented in Fig. 7, and the summarizing of photovoltaic parameters of the devices are estimated in Table 2. Electrical efficiency is a measure the overall power conversion efficiency (PCE) which calculate from the total electrical energy output with the total input energy present in the solar irradiance. The total energy is very close to 100 mW/cm^2^. The junction of ITO/PEDOT:PSS/CuPc/C_60_/Al without AuNPs was used as a reference cell. It was cleared that the short-current density (*Jsc*) was increased with increasing the applied voltage due to the applied voltage increases the mobility, and charge carriers.

**Table 2:** Photovoltaic parameters calculated from J-V characteristic for gold nanoparticles samples (S1-S6) that prepared by adding of (0.5, 0.75, 1.0, 1.25 1.5, and 1.75 ml) from tri-sodium citrate (1%) to boiling of 20 ml of HAuCl_4_·3H_2_O (1.0 mM), respectively and doped in PEDOT: PSS/CuPc/C60/ITO/glass substrate.

Samples	J_SC_ (mA/cm^2^)	V_OC_ (Volt)	J_max_ (mA/cm^2^)	V_max_ (Volt)	FF	Efficiency%
ITO/PEDOT:PSS/CuPc/C60/Al	5.70	0.418	3.90	0.280	0.458	0.78
ITO/PEDOT:PSS+AuNPs (12 nm)/CuPc/C60/Al (S1)	6.61	0.44	4.75	0.300	0.49	1.02
ITO/PEDOT:PSS+AuNPs (14 nm)/CuPc/C60/Al (S2)	6.50	0.436	4.61	0.290	0.472	0.95
ITO/PEDOT:PSS+AuNPs (15 nm)/CuPc/C60/Al (S3)	6.38	0.435	4.46	0.289	0.464	0.92
ITO/PEDOT:PSS+AuNPs (17 nm)/CuPc/C60/Al (S4)	6.29	0.433	4.37	0.288	0.462	0.91
ITO/PEDOT:PSS+AuNPs (19 nm)/CuPc/C60/Al (S5)	6.18	0.431	4.25	0.287	0.458	0.87
ITO/PEDOT:PSS+AuNPs (23 nm)/CuPc/C60/Al (S6)	6.05	0.427	4.14	0.285	0.457	0.84

The values of (*Jsc*) and the open-circuit photovoltage (*Voc*) are extracted at *V* = 0 and *J* = 0 respectively. The maximum current and voltage (*J*_*max*_ and *V*_*max*_) are obtained at the maximum output power point. The fill factor *(FF)* is defined as the ratio of the maximum power from the heterojunction solar cell to the product of *V*_*oc*_ and *J*_*sc*_ (eq. (1)). The overall solar energy conversion efficiency is the maximum power extracted compared to the incident solar power (eq. (2)) [60].

(1) $$\left(FF=J_{\max}\cdot V_{\max}/J_{sc}\cdot V_{oc}\right)$$

(2) $$(PCE=J_{\max}\cdot V_{\max}/P_{in}=(J_{sc}\cdot V_{oc}\cdot FF)/P_{in}$$

*J*_*sc*_, *V*_*oc*_, FF, and efficiency was calculated at 5.7 mA/cm^2^, 0.418 V, 0.458, and 0.78%, respectively of ITO/PEDOT:PSS/CuPc/C_60_/Al without AuNPs. After incorporating the AuNPs into PEDOT:PSS film, all these values were enhanced as shown in Table 2 and *J*_*SC*_ was increased from 5.7 mA/cm^2^ to 6.61, 6.50, 6.38, 6.29, 6.18, and 6.05 mA/cm2, and the power conversion efficiency (PCE) increased from 0.78% to 1.02, 0.95, 0.92, 0.91, 0.87, and 0.84 when PEDOT:PSS was embedded with 12, 14, 15, 17, 19, and 23 nm respectively. This means that, the efficiency increases by 24%, 17%, 14%, 13%, 9%, and 7% when the PEDOT:PSS embedded by AuNPs with sizes 12, 14, 15, 17, 19, and 23 nm respectively. The results distinctly specified that Jsc and PCE were an additional enhanced photovoltaic cell in the presence of AuNPs with different sizes in the PEDOT:PSS film compared to those of the solar cells without plasmonic AuNPs. Our work can be compared with the previous work by Chakaroun et al., [26]. It was found the efficiency in our work was increased due to PEDOT:PSS embedded by AuNPs. Also, this improvement in OPV cell results from the localized surface plasmon effect [61]. By comparing the effect of particles sizes on efficiency it notes that the efficiency increases with reduction of the particle size of AuNPs that are integrated into OPV cell. AuNPs with small sizes produced greater efficiency improvement. This is reasonable because the inbuilt electric field around the small size is higher than that around the large size. This inbuilt electric field increases the dissociation of excitons to electron and hole at interfacial between CuPc and C_60_. In addition, the number of excitons was increased in CuPc layer due to emitted fluorescence from the AuNPs that absorbed by CuPc in the cells [62]. Thus, the increase in the efficiency is due to generated fluorescence that has increased the number in the generation of excitons and the inbuilt electric field herewith increasing the dissociation of these excitons at the interfacial. These two factors increase the electrons and holes in the cathode and anode terminals, thus increasing the efficiency in the OPV cells.

## 4 Conclusion

Gold nanoparticles have been prepared with different sizes and embedded in PEDOT:PSS to enhance the PCE of OPV cell (ITO/PEDOT:PSS (AuNPs)/CuPc/C_60_/Al). J-V characteristics were investigated under and the efficiency was improved after embedded AuNPs into PEDOT:PSS. The maximum efficiency was determined at 1.02% of AuNPs with particle size at 12 nm. The improvement is due to the increase in the photon incident in CuPc and inbuilt electric field at the interfacial between CuPc/C_60_ due to the surface plasmonic resonance. The incident photon in CuPc increases the exciton generation while inbuilt electric field increases the dissociation of these excitons and separated to electrons and holes that affected on the efficiency.

## Declarations

### Author contribution statement

D. A. Said: Conceived and designed the experiments; Analyzed and interpreted the data; Wrote the paper.

A. M. Ali: Conceived and designed the experiments; Performed the experiments; Wrote the paper.

M. M. Khayyat: Conceived and designed the experiments; Performed the experiments; Analyzed and interpreted the data.

M. Boustimi: Conceived and designed the experiments; Contributed reagents, materials, analysis tools or data.

M. Loulou: Conceived and designed the experiments; Wrote the paper.

R. Seoudi: Performed the experiments; Analyzed and interpreted the data; Wrote the paper.

### Funding statement

This work was supported by the Deanship of Scientific Research at Umm Al-Qura University (Project ID 43405060).

### Competing interest statement

The authors declare no conflict of interest.

### Additional information

No additional information is available for this paper.
